# Supplementary material for: Breast Cancer Classification Based on Proteotypes Obtained by SWATH Mass Spectrometry
Source: Cell Rep. 2019 Jul 16;28(3):832–843.e7. doi: 10.1016/j.celrep.2019.06.046 (PMC6656695; doi:10.1016/j.celrep.2019.06.046)
Supplement: Document S1. Figures S1–S6 and Table S1 [file mmc1.pdf]

Cell Reports, Volume 28

Supplemental Information

Breast Cancer Classification Based on Proteotypes Obtained by SWATH Mass Spectrometry

Pavel Bouchal, Olga T. Schubert, Jakub Faktor, Lenka Capkova, Hana Imrichova, Karolina Zoufalova, Vendula Paralova, Roman Hrstka, Yansheng Liu, Holger Alexander Ebhardt, Eva Budinska, Rudolf Nenutil, and Ruedi Aebersold

Supplemental materials

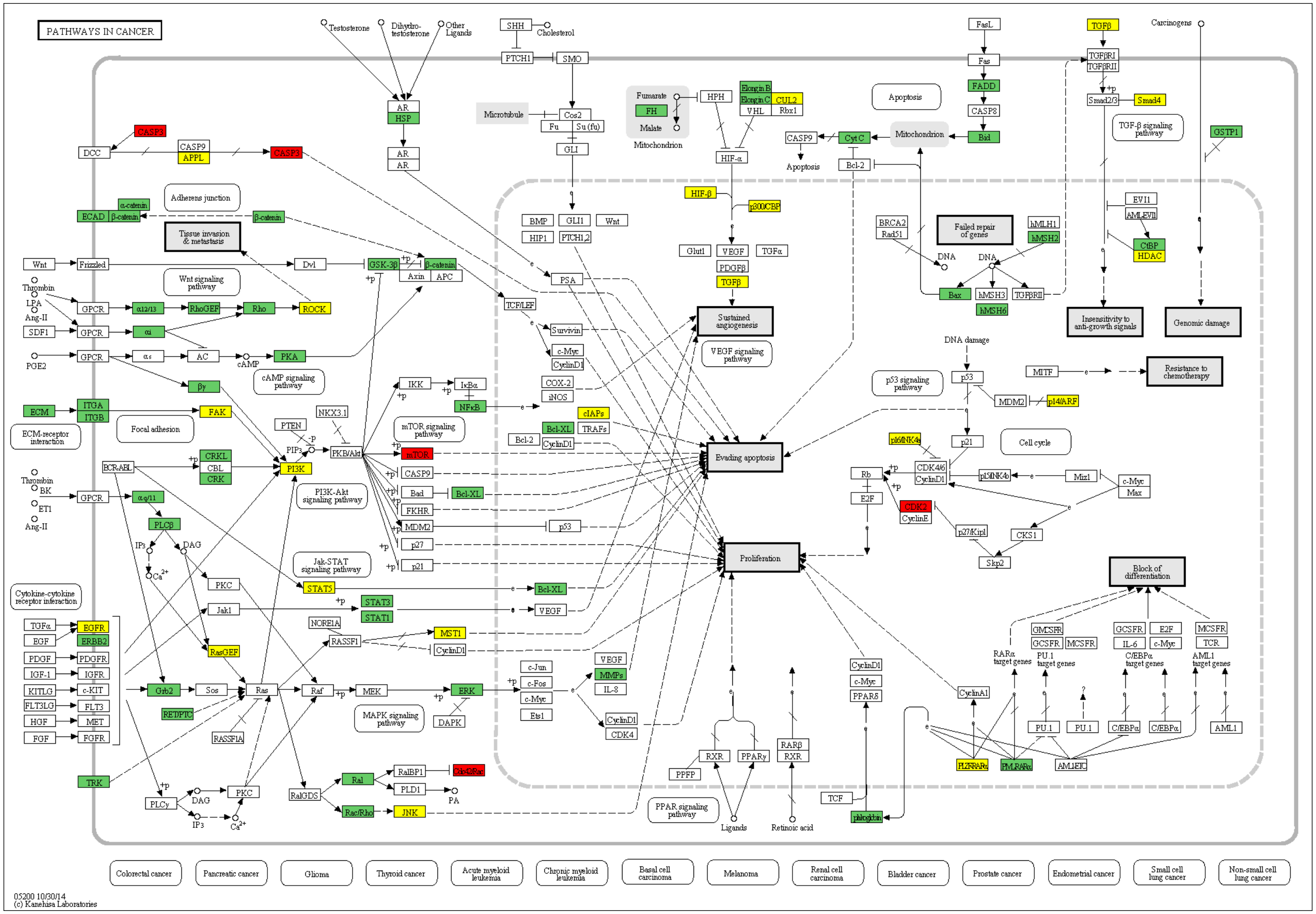

Figure S1. Overlap of cancer-related proteins identified by SWATH-MS and iTRAQ. Related to Figures 1 and 2. The overlap in coverage of cancer-related proteins between SWATH-MS and iTRAQ is shown on KEGG's "Pathways in Cancer" map. The same set of 96 breast cancer tissues was analysed. Yellow boxes correspond to proteins quantified by iTRAQ only (Bouchal et al., 2015), red boxes correspond to proteins quantified by SWATH-MS only, and green boxes correspond to proteins present in both datasets. The FDR cut-off for both methods is 1%. The total number of proteins in the SWATH-MS dataset is 2,842 and the total number of proteins in the iTRAQ dataset is 3,007. Detailed information for the pathway map is available on the KEGG website at www.genome.jp/kegg/.

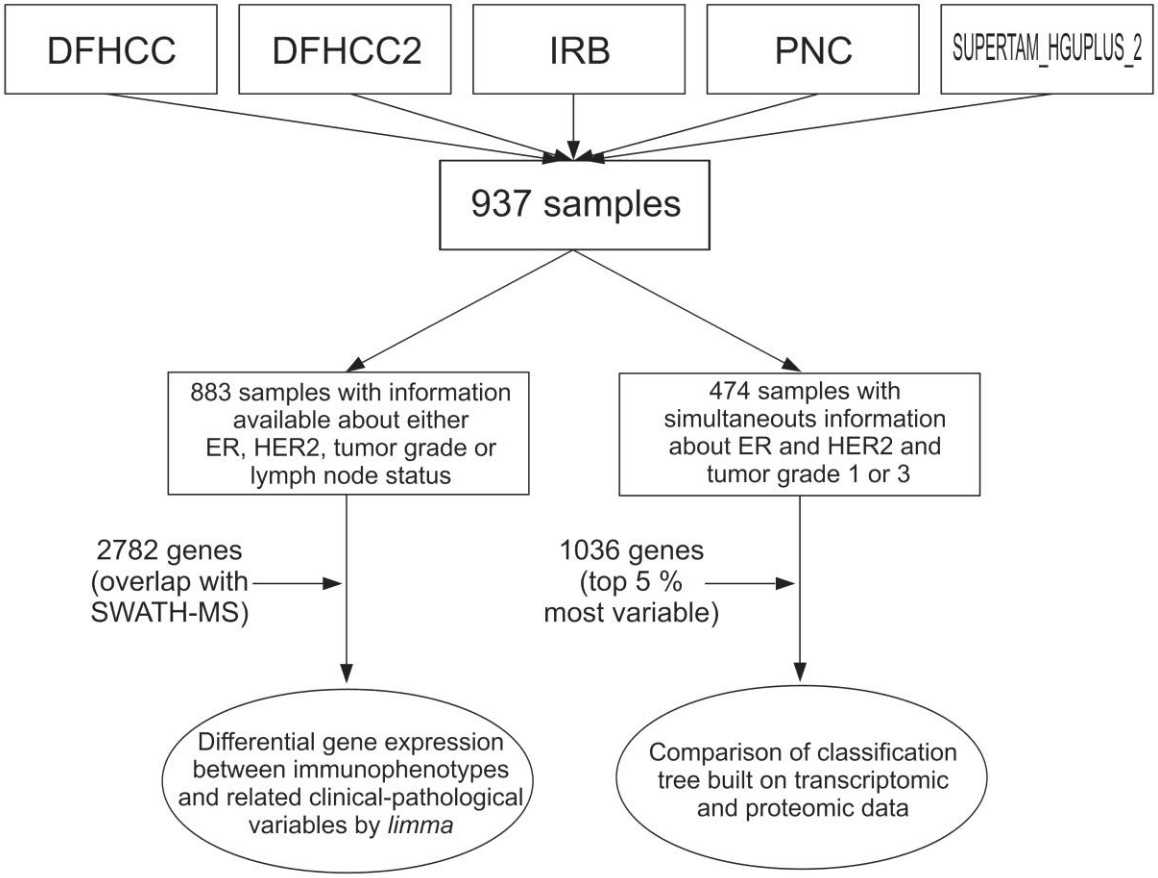

Figure S2. Overview of samples from independent transcriptomics datasets DFHCC, DFHCC2, IRB, PNC and SUPERTAM_HGUPLUS_2 and how they were used for comparisons with the proteomic data and to build a decision tree. Related to Figures 4-6. Data set source: (Haibe-Kains et al., 2012).

A

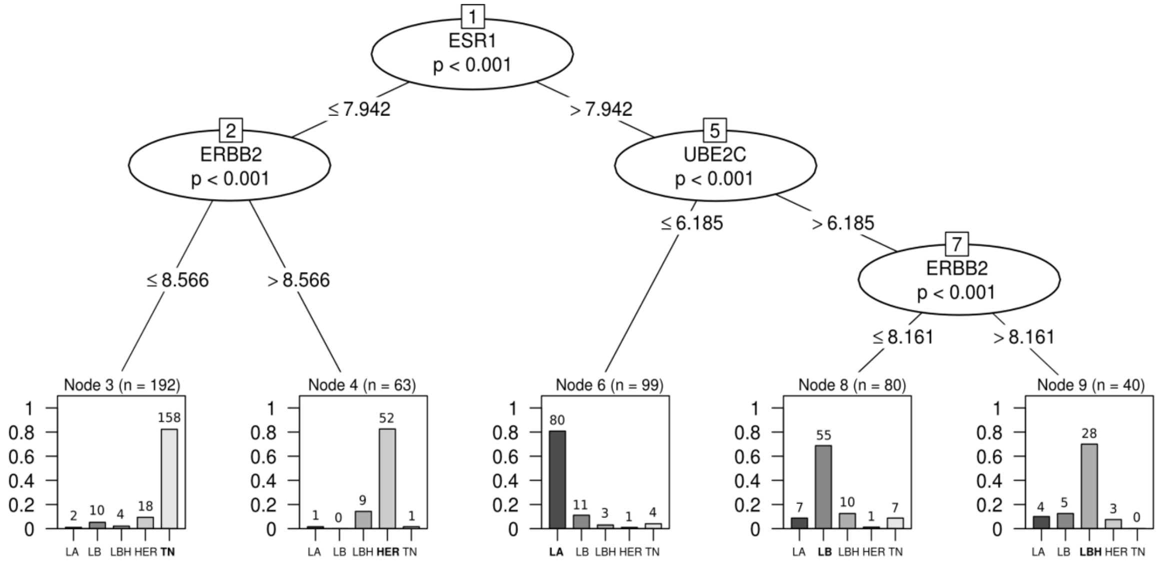

B

		Tree prediction					
		**LA**	**LB**	**LBH**	**HER**	**TN**	**Row sums**
Conventional subtypes of breast cancer	**LA**	**80**	7	4	1	2	94
	**LB**	11	**55**	5	0	10	81
	**LBH**	3	10	**28**	9	4	54
	**HER**	1	1	3	**52**	18	170
	**TN**	4	7	0	1	**158**	170
	**Column sums**	99	80	40	63	192	**474**

Number of correctly classified samples into breast cancer subtypes	373
% of correctly classified samples into breast cancer subtypes	**78.69**

Figure S3. Decision tree based on gene expression data. Related to Figures 3-4. (**A**). Five selected breast cancer gene expression datasets (DFHCC, DFHCC2, IRB, PNC, SUPERTAM_HGU133PLUS2) (Haibe-Kains et al., 2012) were used to construct the decision tree using the same recursive partitioning algorithm as for the proteomic data. See STAR Methods for further details on datasets and analysis. (**B**) Data table for the generation of the decision tree shown in A. Breast cancer subtypes: LA, luminal A; LB, luminal B; LBH, luminal B HER2^{+}, HER, HER2 enriched; TN, triple negative.

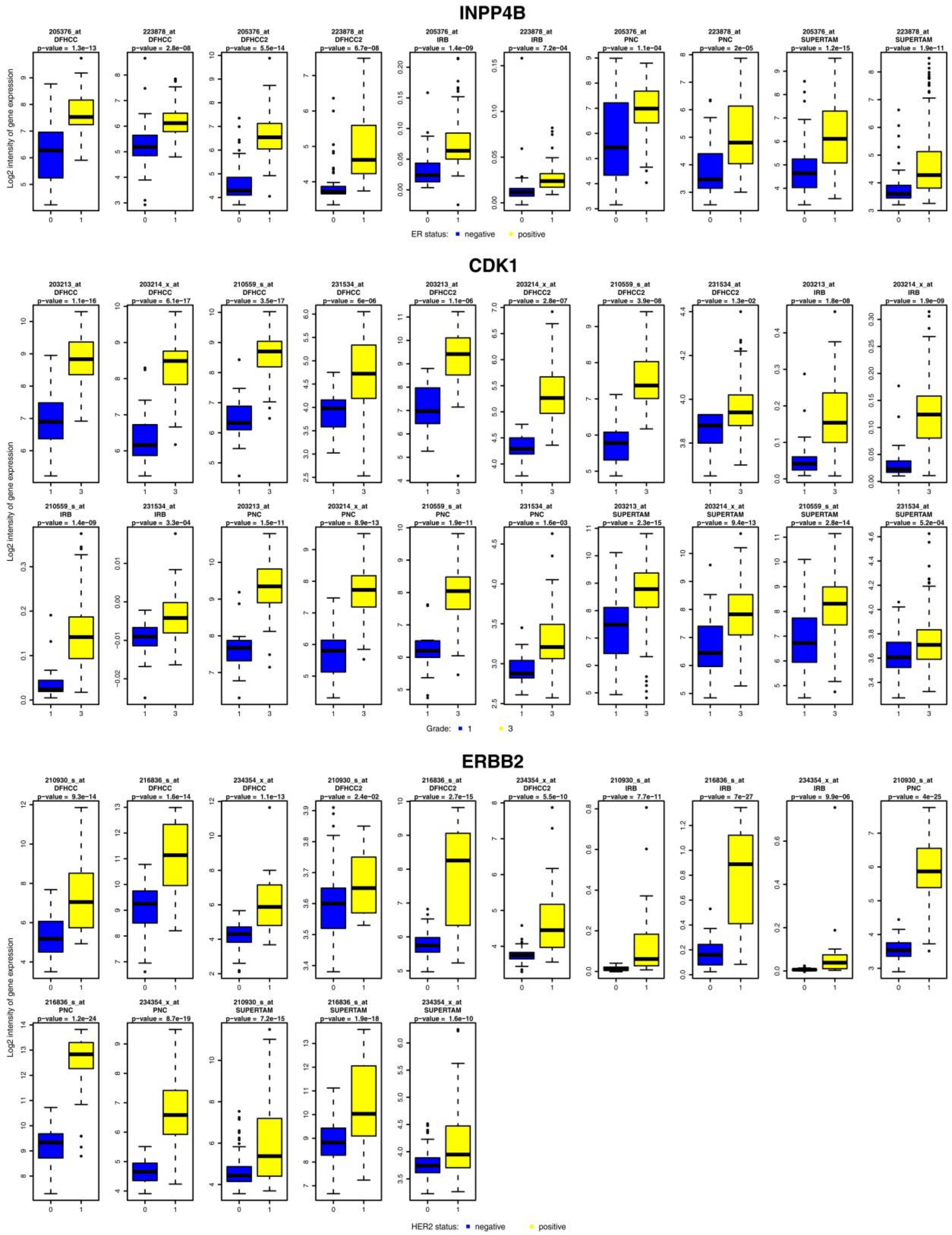

Figure S4. Independent validation of *INPP4B, CDK1* and *ERBB2* association with ER status, tumour grade, and HER2 status (full version). Related to Figure 4. Five independent transcriptomics datasets of 937 patients (DFHCC (n=115), DFHCC2 (n=84), IRB (n=129), PNC (n=92) and SUPERTAM_HGU133PLUS_2 (n=517)) (Haibe-Kains et al., 2012), see STAR Methods and Data file S4D for details) were analysed for gene expression of *INPP4B*, *CDK1*, and *ERBB2*. For each of the three genes, transcript levels were significantly different ($p<0.05$)

depending on ER status (for *INPP4B*), tumour grade (for *CDK1*), or HER2 status (for *ERBB2*). All available Affymetrix probes per gene are shown here; data for the most variable Affymetrix probe are shown in Figure 4.

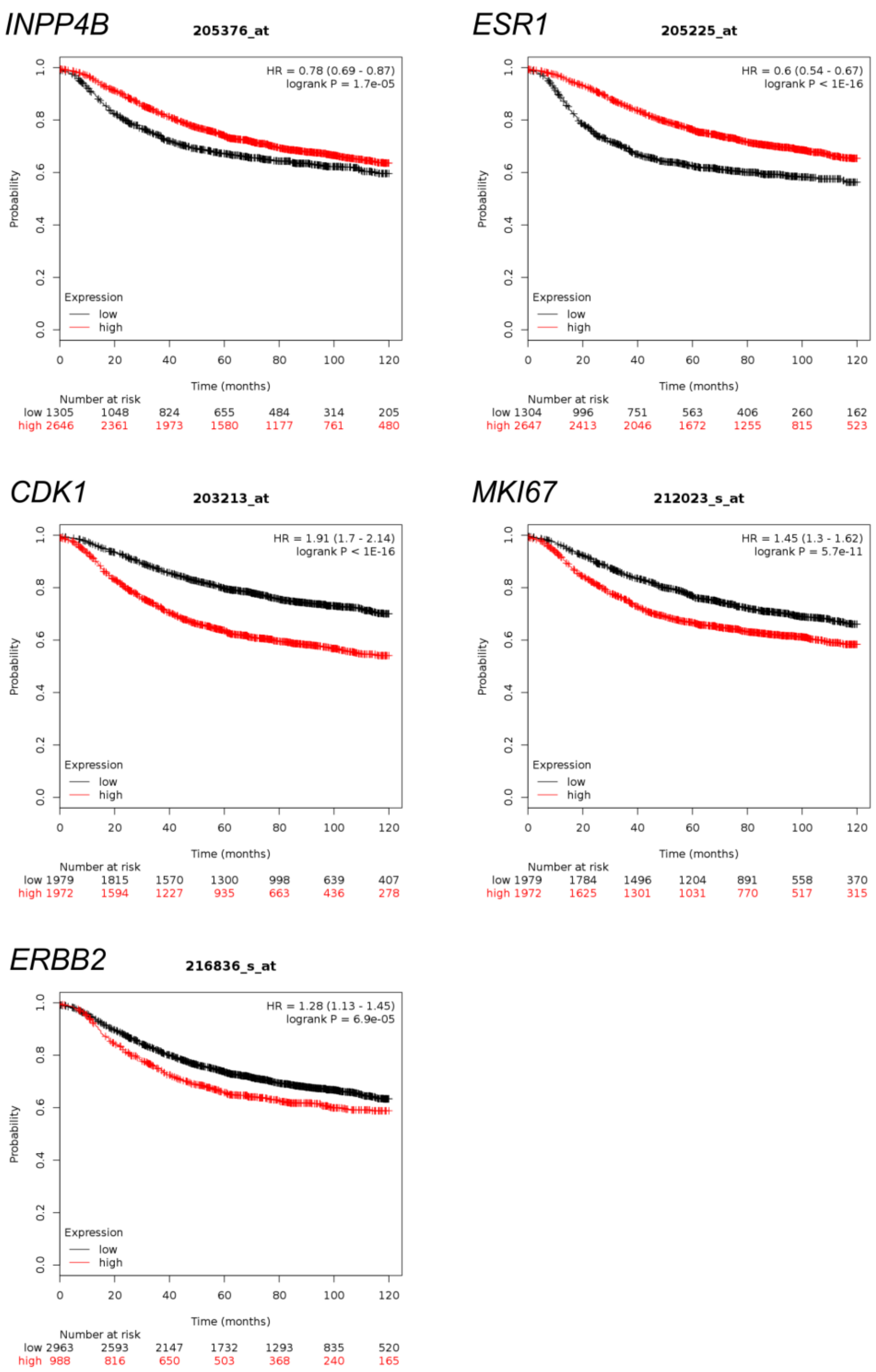

Figure S5. Relapse-free survival (RFS) in breast cancer patients with high vs. low expression of the three key genes *INPP4B, CDK1, ERBB2*. Related to Figures 3-4. A microarray dataset of 3955 breast cancer patients available through the KMplot tool (www.kmplot.com, 2018 database version) was used to generate Kaplan-Meier plots. Selected cut-offs for high vs. low expression correspond to representation in breast cancer patient population. We found that *INPP4B* expression was significantly connected with RFS in the same manner as the commonly used reference gene *ESR1* (for ER status), and that *CDK1* expression was significantly connected with RFS in the same manner as the commonly used reference gene *MKI67* (for tumour proliferation/grade).

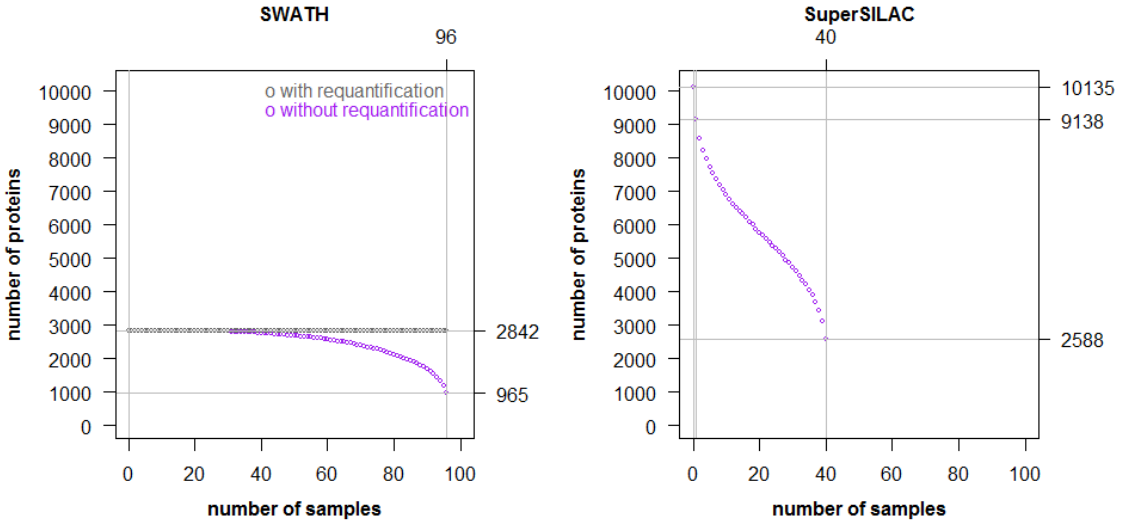

Figure S6. Comparison of the number of proteins consistently quantified across samples by SWATH-MS and SuperSILAC. Related to Figures 1 and 3. Plots show the number of proteins consistently quantified across samples as a function of samples analysed. Shown on the left is the SWATH-MS dataset of 96 patients and on the right the SuperSILAC dataset of 40 patients (Tyanova et al., 2016). Note that the comparison is based only on light peptide variants originating from tissue; since the labeled ("heavy") internal standard peptides used in the SuperSILAC study were prepared from cell lines and spiked into the tissue sample, they are not relevant for the number of tissue proteins quantified. „Requantification" refers to a software tool that is run as part of the SWATH-MS data analysis pipeline for peptides not detectable above an FDR of 0.01. In these cases, the tool infers the peak boundaries from the closest neighboring run and quantifies the background fragment ion signal within those boundaries. These imputed values serve as upper bounds of the analyte signal for the run in question (Rost et al., 2016).

		Abundance rank	
Gene symbol	UniProt Acc.	Bouchal *et al.*	Tyanova *et al.*
HER2	P04626	997	197
GRB7	Q14451	959	1,080
MAPK3	P27361	1,302	1,775
EEF1G	P26641	168	156
AGR2	O95994	291	212
NDUFAB1	O14561	2,149	2,338
MIPEP	Q99797	-	2,820
MLPH	Q9BV36	2,396	2,322
MCM5	P33992	1,481	1,486
HID1	Q8IV36	1,275	2,567
STMN1	P16949	460	1,123
CMBL	Q96DG6	1,456	1,124
GLS	O94925	2,536	2,031
FOXA1	B7ZAP5	-	3,574
C9orf114	Q5T280	-	4,924
RCL1	Q9Y2P8	-	2,871
ECM1	Q16610	1,323	2,552
CAPN7	Q9Y6W3	-	3,998
ENO1	P06733	26	28
		of 2,842	of 10,138

Table S1: Coverage and abundance ranks of 19-protein signature identified by SuperSILAC compared to the abundance rank of the same proteins in our SWATH-MS dataset. Related to Figure 1. Abundance ranks of 19 signature proteins identified by Tyanova and colleagues (Tyanova et al., 2016) presented in their Fig. 6A were compared to their abundance ranks in our study. Of the 19 key proteins, 14 were quantified in our study with a similar abundance rank.
